# 644. Disparities in Screening and Test Positivity for Chlamydia and Gonorrhea Among Adolescent and Young Adult Women in a Massachusetts Integrated Healthcare System, 2016–2024

**DOI:** 10.1093/ofid/ofaf695.208

**Published:** 2026-01-11

**Authors:** Sarah Naz-McLean, Lisa A Cosimi, Ann E Woolley, Ann Burchell, Amaya Perez-Brumer

**Affiliations:** Brigham and Women's Hospital, Boston, Massachusetts; Brigham & Women's Hospital, Boston, Massachusetts; Brigham and Women's Hospital, Boston, Massachusetts; Unity Health, Toronto, Ontario, Canada; University of Toronto, Toronto, Ontario, Canada

## Abstract

**Background:**

Young women in the U.S. aged 15–24 bear among the highest burden of chlamydia (CT) and gonorrhea (NG) infections. Despite national guidelines recommending annual screening for women under 25, screening uptake remains suboptimal. This study examines demographic patterns in CT/NG testing and positivity to understand how access to different care settings shapes testing uptake to inform routine sexual health screening strategies.

**Methods:**

Data are derived from cisgender women and adolescents aged 15–29 tested for CT/NG within the Mass General Brigham integrated healthcare system between January 2016 and January 2024. Laboratory records were linked to the closest clinical encounter within 7 days. Descriptive statistics summarized test volume, positivity, and diagnoses by demographics and care setting. Bivariate and multivariable Poisson GEE models estimated the risk ratios (RR) between demographic variables and the outcomes of 1) being tested in acute care (ED, Urgent Care, walk-in) vs. all other care settings and 2) symptomatic vs. asymptomatic presentation (via ICD-10 codes for urogenital symptoms).

**Results:**

Of 134,607 unique testing episodes (median age 24), CT test positivity was 3.0% and NG positivity 0.3%. Most tests occurred in primary care (46.2%) and OB-GYN settings (33.8%); 5.0% occurred in ED and 2.5% urgent care/walk-in settings. CT positivity was highest among women and adolescents aged 15-19, Black, Hispanic, and uninsured or on public insurance. Black (aRR 1.92, 95%CI: 1.79, 2.07), Hispanic (aRR 1.71, 95%CI: 1.59, 1.84), and uninsured women (aRR 2.23, 95%CI: 1.90, 2.63), and those aged 15-19 were more likely to test in acute care settings. Symptomatic presentation was more likely among Black (aRR 1.32, 95% CI: 1.27, 1.37); Hispanic (aRR 1.31, 95%CI: 1.26, 1.36); and uninsured women (aRR 1.59, 95% CI: 1.46, 1.74); and those with mixed public/private insurance (aRR 1.38, 95% CI: 1.22, 1.55).

**Conclusion:**

Conclusions: Higher test positivity, symptomatic presentation, and acute care use among younger, black, Hispanic, and uninsured women and adolescents suggest inequities in access to non-acute sexual health screening. These findings highlight the need for targeted, culturally responsive strategies to expand preventive CT/NG services.

**Disclosures:**

All Authors: No reported disclosures

